# Factors Linking Interoception and Sleep Across the Adult Lifespan

**DOI:** 10.1111/psyp.70120

**Published:** 2025-08-06

**Authors:** Ahhyun Seo, Risako Nishiyama, Kyoungeun Lee, Audrey Duarte

**Affiliations:** ^1^ Department of Psychology University of Texas at Austin Austin Texas USA

**Keywords:** actigraphy, anxiety, insula, interoception, sleep, voxel‐based morphometry analyses

## Abstract

Sleep patterns change with age, and interoception—a multifaceted concept referring to the perception of internal body sensations—may be one of the underlying mechanisms of these changes. The insula cortex, a key region for both interoception and sleep, might be a shared neural link. In this study, we examined the role of the insula in linking multiple interoceptive constructs with objectively measured sleep. We also investigated how interoception relates to sleep quality across the adult lifespan at different levels of anxiety. We measured interoceptive accuracy (the objective ability to detect internal signals), interoceptive sensibility (the self‐perceived ability to detect these signals), insular volume (using structural MRI), objectively assessed sleep quality (via actigraphy), and trait anxiety in 70 participants aged 18–79. The results indicated that both interoceptive constructs were associated with poorer sleep quality across age, particularly in individuals with higher anxiety levels. We also found that greater insula volume was associated with a stronger subjective belief in one's interoceptive abilities (interoceptive sensibility). Although there was no direct link between insula volume and sleep quality, insula integrity may indirectly influence sleep quality through its association with interoceptive sensibility. These findings highlight the negative relationship between interoception and sleep quality across the adult lifespan. Sleep interventions using mindfulness‐ or interoception‐focused strategies should be implemented with caution, particularly for anxious individuals or those with heightened interoceptive sensibility. Further research should include poor sleepers and individuals with a wide range of health conditions for better understanding.

## Abstract 2

This study examined interoceptive constructs and sleep quality across adulthood, focusing on the insula's role in these relationships. Results suggest that both actual and perceived interoceptive abilities are related to sleep quality, particularly in individuals with high anxiety, across all ages. The insula cortex is closely associated to perceived interoceptive ability and may indirectly relate to sleep quality. Sleep interventions should be tailored to consider specific interoceptive traits and individual anxiety levels.

## 1 Introduction

Sleep patterns significantly change with aging. Older adults tend to experience more frequent awakenings during the night (Bliwise et al. 2009), shorter sleep durations, and decreased sleep efficiency (Ohayon et al. 2004), compared to younger adults. These sleep difficulties have been associated with cognitive impairments including deficits in memory (as reviewed in Stickgold and Walker 2013) and executive functions (Holanda Júnior and Almondes 2016), emotional problems such as depression and anxiety (Mallon et al. 2000; Yu et al. 2016), and age‐related pathologies like Alzheimer's disease (Lucey et al. 2019). Several mechanisms underlying poor sleep quality in aging have been explored, including alterations in circadian rhythm (Wright and Frey 2008), related hormones such as melatonin (as reviewed in Pandi‐Perumal et al. 2005), cortisol (Buckley and Schatzberg 2010), and thyroid‐stimulating hormone (as reviewed in Copinschi and Caufriez 2013; Li, Dong, et al. 2018; Li, Vitiello, and Gooneratne 2018), age‐related reductions in brain integrity (Branger et al. 2016; Liu et al. 2018), and various medical conditions (as reviewed in Avidan and Alessi 2008).

Interoception, the perception of internal body sensations such as heartbeat, breathing, body temperature, and stomach sensations (Craig 2002), has been gaining attention for its potential link to sleep. Internal physiological systems function in circadian rhythms that are linked to the sleep–wake cycle (as reviewed in Chokroverty 2017; Trinder et al. 2012), as reflected in changes in cardiovascular activity (as reviewed in Trinder et al. 2012; Carrington et al. 2005; Idema et al. 1992), body temperature (Harding et al. 2019), respiration (Guilleminault et al. 2001; as reviewed in Mortola 2004; Spengler et al. 2000), and gastrointestinal function (as reviewed in Konturek et al. 2011; Voigt et al. 2019) throughout the day. Perceiving these internal changes may facilitate sleep onset or help prevent disruptions during sleep (Harding et al. 2019; as reviewed in Wei and Van Someren 2020). Yet, the relationship between interoception and sleep appears complex as prior evidence indicated that heightened interoception is also linked to insomnia symptoms in both general and clinical populations (Hammad et al. 2001; Sunnhed and Jansson‐Fröjmark 2015; Wei et al. 2016). Insomnia is characterized by autonomic dysregulation and hyperarousal (as reviewed in Perlis et al. 1997; Pigeon and Perlis 2006; Perlis et al. 2011; Riemann et al. 2010). This hyperarousal may be linked to increased attention to internal signals as they become more prominent (i.e., Wei et al. 2016).

In the context of aging, there is reason to predict that interoception may be impacted by age, which, in turn, may impact sleep quality. For instance, interoception is linked to various health factors that are also associated with aging including heart rate variability (HRV; Knapp‐Kline and Kline 2005; Lischke et al. 2021), body composition (Murphy et al. 2018), cognitive impairment (Haustein et al. 2023), and reduced insula cortex volume (Fermin et al. 2023; Longarzo et al. 2021). However, the relationship between age and interoception remains unclear due to mixed findings. Some studies have shown that interoception decreases with age (Khalsa et al. 2009; Murphy et al. 2018), whereas others have found no relationship (Raimo et al. 2021; Mikkelsen et al. 2019; Haustein et al. 2023) or even an increase (Stevenson et al. 2018). These discrepancies may arise from inconsistent interoceptive conceptualizations, failing to account for its multifaceted nature (Desmedt et al. 2023; Fittipaldi et al. 2020).

Several multi‐construct models of interoception have been proposed (i.e., Garfinkel et al. 2015; as reviewed in Khalsa and Lapidus 2016). The most studied construct is interoceptive accuracy, which reflects how accurately an individual can detect physiological signals such as those from the cardiovascular system (Desmedt et al. 2023; Garfinkel et al. 2015). Interoceptive accuracy is typically measured using behavioral tests (e.g., the heart tapping task: Comparing the number of heartbeats perceived by an individual with the actual number measured; Smith et al. 2021). Another construct of interoception is interoceptive sensibility, which refers to the self‐assessed ability to detect bodily sensations, often measured through self‐report questionnaires (Desmedt et al. 2023; Garfinkel et al. 2015). Interoceptive sensibility does not necessarily correlate with interoceptive accuracy. However, it captures bodily sensations in a more general way rather than focusing on a specific physiological system (e.g., “I can return awareness to my body if I am distracted,” “I notice how my body changes when I feel happy/joyful”). As interoceptive accuracy and sensibility address different objective and subjective aspects of interoception, respectively, it has been suggested that both should be considered for a more comprehensive understanding of interoception (Garfinkel et al. 2015; Lenggenhager et al. 2013).

A handful of studies have examined the relationship between interoception and habitual sleep quality. However, these studies are constrained by the limited application of interoceptive constructs and a lack of consideration for age‐related differences. For instance, LoBrutto (2024) investigated the relationship between interoceptive sensibility using the Multidimensional Assessment of Interoceptive Awareness Version 2 (MAIA‐2), a self‐report questionnaire with eight subscales, and self‐reported sleep quality. The study showed that higher scores in the sum of six subscales of MAIA‐2, though not all, were related to better self‐reported sleep quality. In the study by Arora et al. (2021), higher scores in some subscales of MAIA‐2 (non‐distracting and trusting) were associated with better sleep quality, whereas higher scores in some other subscales (noticing and emotional awareness) were linked to poorer sleep quality. Ewing et al. (2017) found that greater interoceptive sensibility measured with the “awarenes” subscale from the Porges Body Perception Questionnaire (Porges 1993) was related to increased self‐reported sleep difficulties.

One potential explanation for the mixed findings of the association between interoception and sleep quality is related to the role of affective symptoms that are seldom measured but may vary between individuals and study samples across studies. For example, trait anxiety has been associated with heightened interoceptive accuracy (as reviewed in Domschke et al. 2010; Paulus and Stein 2010; Richards et al. 2003). Of the few studies investigating the association between interoception and sleep, one examined multiple interoceptive constructs and self‐reported sleep quality in individuals with mental health diagnoses (e.g., major depression, anxiety, psychosis, bipolar disorder) and healthy controls (Ewing et al. 2017). In this study, reduced interoceptive accuracy was associated with increased self‐reported sleep difficulties, particularly in those diagnosed with anxiety and depressive disorders, whereas greater interoceptive sensibility was linked to increased sleep difficulties across groups. These results suggest that anxiety and depression may moderate the relationship between interoception and sleep, though it should be noted that this study did not assess the continuum of anxiety and depression levels and focused on clinically diagnosed participants. Pollatos et al. (2009) argued that the relationship between depression and interoception was significant only in individuals with high anxiety, suggesting that anxiety, in particular, may play a role in modulating attention to interoceptive signals. Given this particular relationship between anxiety and interoception, the current study focused on anxiety's potential moderating role between interoception and sleep.

Neuroimaging studies highlight the role of the insula cortex in both sleep and interoception. The insula cortex is primarily involved in visceral‐somatic processing, autonomic control, and socioemotional processing (Uddin et al. 2017). Previous research with healthy samples indicated that smaller insula volume was associated with greater self‐reported sleep disturbances in middle‐aged to older adults (Branger et al. 2016) and decreased interoceptive sensibility in young to middle‐aged adults (Longarzo et al. 2021) and interoceptive accuracy in middle‐aged females (Fermin et al. 2023). Given that insula volume significantly declines with age (Bergfield et al. 2010), it is possible that age‐related declines in insula integrity may contribute to decreases in interoception and sleep quality, although this has yet to be fully investigated.

To date, no studies have yet examined the relationship between different interoceptive constructs and objective sleep quality across the adult lifespan, nor the insula's role in potentially linking interoception and sleep. Given that self‐reports of sleep quality can be inaccurate, especially in individuals with sleep difficulties like many older adults (Girschik et al. 2012; Ma et al. 2021), there remains a significant gap in understanding the interoception‐sleep connection as previous studies primarily used self‐report measures. Additionally, it has been noted that most people are unable to perceive their cardiac sensations under physiological rest. Thus, inducing homeostatic perturbations has been suggested to improve the construct validity of interoceptive accuracy (Khalsa et al. 2008; Khalsa and Lapidus 2016). In this study, we investigated the role of insula cortical volume in both interoception (accuracy and sensibility) and sleep, as well as how interoception relates to sleep across the adult lifespan at different levels of anxiety. Specifically, we examined interoceptive accuracy under perturbation conditions (breath‐holding) to enhance interoceptive signal detection, alongside objective sleep patterns using actigraphy in a healthy adult lifespan sample. We also assessed the level of anxiety on a continuum as a potential moderator in the relationship between interoception and sleep. Our research questions and predictions were as follows:

1. We examined whether insula volume and age were associated with objectively measured sleep quality and interoception across various constructs. We predicted that a smaller insula volume would be related to poorer sleep quality and decreased interoception (Bergfield et al. 2010; Branger et al. 2016; Longarzo et al. 2021). We did not make a strong prediction about the relationship between age and interoception given the limited previous research investigating multiple interoceptive constructs in relation to aging.
2. We examined the relationship between interoceptive constructs and objectively measured sleep quality, and how these relationships varied as a function of anxiety level. Although there are limited and inconsistent findings from previous studies, we predicted that decreased interoception would be related to poorer sleep quality based on some of the existing evidence (Ewing et al. 2017; LoBrutto 2024). Additionally, we hypothesized that anxiety would moderate these relationships, such that higher levels of anxiety would weaken or even reverse the positive associations. Greater anxiety may be linked to heightened physiological arousal including faster heart rate (as reviewed in Coles et al. 2015). If individuals with high anxiety are also highly interoceptive, they may be more attentive to this heightened arousal, which could further disrupt sleep. In this study, anxiety levels were measured on a continuum, focusing on individual differences within a healthy sample.

## 2 Methods

### 2.1 Participants

This study was approved by the Institutional Review Board at the University of Texas at Austin. Eighty healthy adults between the ages of 18 and 79 without any neurological, cardiovascular, psychiatric, and sleep disorders were recruited. All participants were right‐handed. Of the 80 participants, 4 were excluded (1 participant due to incidental findings in the neural images, and 3 participants were outliers for the sleep measures—±3 SD from the mean—see Section 2.3.3). Five participants were missing sleep data due to nonadherence with the accelerometer. One participant had missing data for the interoceptive measures. The demographics of participants are shown in Table 1.

**TABLE 1:** Demographics of participants.

Measures	Final sample (*N* = 70)
Age	48.5 (±18.32)
Years of education	17.71 (±2.31)
*Biological sex (n, %)*	
Female	40 (57.1%)
Male	30 (42.9%)
*Racial/ethnic identity (n, %)*	
Non‐Hispanic White	48 (68.6%)
Black	1 (1.4%)
Asian	13 (18.6%)
Hispanic	4 (5.7%)
Other	4 (6.7%)

### 2.2 Procedure

There were two lab visits with a week between each visit. The week gap was to impose a memory delay, which will not be discussed in this paper. During their first lab visit, neural imaging data were acquired with a structural MRI (3T Siemens Vida scanner at the Brain Imaging Center at UT Austin). Participants were also given an accelerometer (Philips Actiwatch 2) to collect 7 days of sleep data between their first and second visits.

For their second visit, participants were first asked to stand on a body composition analyzer (Seca Technologies, Chino, CA, USA), which measured body mass index (BMI) and fat mass index (FMI) (see Section 2.3.5). Then, participants were seated in a private testing room and completed self‐report questionnaires: the Multidimensional Assessment of Interoceptive Awareness (MAIA‐2; Mehling et al. 2018; see Section 2.3.2) and the State–Trait Anxiety Inventory for adults (STAI; Spielberger et al. 1983; see Section 2.3.4). After completing the questionnaires, participants had Ag‐Cl electrodes (EL503; BIOPAC Systems) attached on the right clavicle and beneath the right and left lower ribs to collect electrocardiographs (ECG; BIOPAC Systems, Santa Barbara, CA, USA). After recording baseline heartrate for 5 min, we continued to record ECG while participants completed an interoceptive behavioral task—the heartbeat tapping task—in which they were asked to press a key each time they perceived their heartbeat (Smith et al. 2021; see Section 2.3.1).

### 2.3 Measures

#### 2.3.1 Interoceptive Accuracy (IAcc)

Interoceptive accuracy, the objective perception of bodily sensations (Garfinkel et al. 2015), was obtained through a heartbeat tapping task, following the procedures from Smith et al. (2021). Participants were asked to press the down key on the keyboard each time they felt their heartbeat over a duration of 60 seconds. ECG was also recorded while participants completed the heartbeat tapping task, to compare the timing of their actual heartbeats to when they pressed the down key (BIOPAC Systems, Santa Barbara, CA, USA). The task was completed under two conditions: the baseline and breath‐holding perturbation condition. During the baseline condition, participants were asked to detect their heartbeats while closing their eyes. The perturbation condition required participants to perceive their heartbeats while holding their breath as long as possible during the 60 seconds. Prior studies suggest using the perturbation condition to improve construct validity by amplifying cardiac signals to better detect heartbeats (Khalsa and Lapidus 2016). Both conditions were repeated twice to increase the reliability of the measure. We used the performance from the perturbation condition to calculate interoceptive accuracy. Interoceptive accuracy was calculated for both trials with this following formula which compares the number of heartbeats from the ECG data to the number of taps made during the task (Smith et al. 2021):

$$\;\text{IAcc}=\frac{\text{Number of heartbeats}-|\text{Number of heartbeats}–\text{Number of taps}\;|}{\text{Number of heartbeats}}$$

We then calculated the averages between the two trials, which was the measure for interoceptive accuracy we used for data analysis. The accuracy measure between the two trials were highly correlated (*r* = 0.85, *p* < 0.001), suggesting high internal reliability.

#### 2.3.2 Interoceptive Sensibility (IS)

Interoceptive sensibility, the self‐perceived ability to detect bodily states (Garfinkel et al. 2015), was measured with the 2nd version of the Multidimensional Assessment of Interoceptive Awareness questionnaire (MAIA‐2; Mehling et al. 2018). The MAIA‐2 consisted of 37 statements pertaining to the individual's belief of their ability to perceive and control their bodily sensations (i.e., “I notice changes in my breathing, such as whether it slows down or speeds up”). Participants were asked to rate how well these statements apply to them in their daily lives on a scale from 0 (“Never”) to 5 (“Always”). Questions were grouped into eight various subscales, which were then calculated by finding the average of responses (ranging from a score of 0 to 5). Since the subscales measure distinct constructs, it is not recommended to use the total score of MAIA‐2 (Vig et al. 2022). The subscales are *noticing* (the ability to detect body sensations), *not‐distracting* (the ability to distract oneself from pain and discomfort in the body), *not‐worrying* (the tendency to not worry when feeling pain or discomfort in the body), *attention regulation* (the ability to maintain attention towards body sensations), *emotional awareness* (awareness of the body sensations associated with emotional states), *self‐regulation* (using body sensations to regulate emotional states), *body listening* (using body sensations to assess emotions and needs), and *trust* (perceiving the body and its sensations as safe and trustworthy).

To reduce the dimensionality of data and simplify subsequent analyses, we conducted principal component analysis (PCA) with varimax rotation with the eight subscales. Principal components with eigenvalues over 1 were kept in the model. We found the same three factors identified in a confirmatory factor analysis (CFA) model in Ferentzi et al. (2021), specifically a general component (comprised of noticing, attention regulation, emotional awareness, self‐regulation, body listening, and trust) and not‐distracting and not‐worrying as separate components. The component loadings are shown in Table 2. In further analyses, the general component was used as the measure of Interoceptive Sensibility as it contained the majority of explained variance in the PCA model. The general component included 43.6% of the explained variance, whereas the not‐worrying and not‐distracting components included 18.9% and 13.5% of the explained variance, respectively, adding up to a cumulative explained variance of 76% in the model.

**TABLE 2:** Component loadings for MAIA‐2 subscales.

MAIA‐2 subscales	General sensibility	Not‐worrying	Not‐distracting
Body listening	**0.836**	−0.013	0.043
Self regulation	**0.820**	0.297	−0.093
Noticing	**0.807**	−0.055	0.033
Emotional awareness	**0.762**	0.189	−0.195
Attention regulation	**0.689**	**0.558**	−0.184
Trusting	**0.640**	**0.475**	0.188
Not worrying	0.023	**0.922**	−0.011
Not distracting	−0.044	−0.010	**0.981**

*Note:* General component explained variance: 43.6%; not‐worrying component explained variance: 18.9%; not distracting component explained variance: 13.5%. Model fit (*ꭓ* ^2^ = 51.34, df = 7, *p* < 0.001). Significance of the bold numbers indicating those with the strongest loadings within a component (≥ 0.40).

#### 2.3.3 Sleep Variables and Principal Component Analysis

We extracted six sleep variables and computed the means from 7 days of actigraphy data: wake after sleep onset (WASO—the total number of minutes awake after initial onset of sleep), total sleep time (TST—minutes asleep), sleep efficiency (percentage of time asleep while in bed), onset latency (the total number of minutes taken to initially fall asleep), Sleep Fragmentation Index (SFI—the sum of percentages of physical movement during the sleep period, and sleep bouts with lower physical movement that are less than 60 s long), and number of awakenings.

To reduce the dimensionality of the actigraphy sleep data, we conducted principal component analysis (PCA) with varimax rotation with the mean values of the sleep variables as in previous studies (Hokett and Duarte 2019; Hokett et al. 2022; Staples et al. 2019). PCA is an effective method for reducing the dimensionality and number of comparisons for multidimensional data in a data‐driven manner (Daffertshofer et al. 2004). Principal components with eigenvalues over 1 were kept, forming two main sleep components used in further analyses: defined as sleep duration/efficiency (TST, sleep efficiency, onset latency) and sleep restlessness (sleep fragmentation, number of awakenings, WASO). The component loadings table is shown in Table 3, with the bold numbers indicating those with the strongest loadings within a component (≥ 0.40).

**TABLE 3:** Component loadings for sleep variables.

Sleep variables	Sleep restlessness	Sleep duration/efficiency
WASO	**0.929**	−0.035
Number of awakenings	**0.903**	0.101
Sleep fragmentation	**0.806**	−0.370
Sleep efficiency	**−0.572**	**0.768**
Onset latency	−0.037	**−0.798**
TST	−0.033	**0.767**

*Note:* Sleep restlessness explained variance: 44.3%; sleep duration/efficiency component explained variance: 32.7%. Model fit (*ꭓ* ^2^ = 58.932, df = 4, *p* < 0.001).

#### 2.3.4 Anxiety

Anxiety was measured with the State–Trait Anxiety Index questionnaire (STAI; Spielberger et al. 1983). The STAI asks participants to rate their feelings and emotions from a scale of 1 (“Not at all”) to 4 (“Very much”). The questionnaire consists of two scales: the state subscale that measures momentary feelings, and the trait subscale that measures general tendencies of anxious feelings. The sum of scores range from 20 (lowest) to 80 (highest). We aimed to examine how individual differences of general anxiety on a continuum would affect the relationship between sleep and interoception. Given that our other measures consist of trait measures such as habitual sleep quality and interoceptive sensibility, we decided to focus on the trait anxiety subscale rather than state anxiety as a moderator between interoception and sleep.

#### 2.3.5 Health Variables

We assessed several health variables to measure their potential impact on the relationship between interoception and aging, as noted in the introduction (Knapp‐Kline and Kline 2005; Murphy et al. 2018; Lischke et al. 2021). Health variables we included in our correlation table were body mass index (BMI), fat mass index (FMI), and short‐term heart rate variability (HRV). BMI and FMI were measured with a full body composition analyzer (mBCA; Seca Technologies, Chino, CA, USA). The mBCA measured fat mass, body weight, and height. BMI was calculated as body weight (kg) divided by height (m^2^), and FMI was calculated as fat mass (kg) divided by height (m^2^). HRV was measured during the first ECG recording trial, which detected baseline heart rate for 300 seconds. We used the AcqKnowledge HRV analysis software (BIOPAC systems, Santa Barbara, CA, USA) to detect the cardiac peaks and conduct time‐domain analysis to measure variability of time in between heartbeats (Shaffer and Ginsberg 2017). The HRV measure we focused on was the root mean square of successive differences between normal heartbeats (RMSSD), measured in milliseconds (ms).

#### 2.3.6 Structural MRI Acquisition and Analysis

A Siemens 3T VIDA‐MRI scanner equipped with a 20‐channel head coil at the UT Austin Brain Imaging Center was used for scanning. A high‐resolution T1‐weighted magnetization‐prepared rapid acquisition gradient‐echo (MPRAGE) image was also collected for normalization (TR = 2300 ms, TE = 2.98 ms, flip angle = 9°, field of view (FOV) = 256 mm, 1 mm isotopic voxels, no gap).

Preprocessing of structural imaging data were performed using the Computational Anatomy Toolbox version 12.7 (CAT12; Gaser et al. 2022) for SPM12 (Wellcome Trust Centre for Neuroimaging, London, UK; https://www.fl.ion.ucl.ac.uk/spm) under MATLAB R2019b (MathWorks, Natick, MA, USA; https://www.mathworks.com). For voxel‐based morphometry (VBM) analyses, the random noise of the T1‐weighted MRI scans was removed using a spatial adaptive non‐local means (SANLM) denoising filter (Manjón et al. 2010), which adjusts the filtering strength based on the local noise level in the image and averages pixels across the entire image with similar intensity patterns, whereas preserving the edges of structures. Then, internal resampling was conducted to accommodate low‐resolution images and anisotropic spatial resolutions, followed by bias correction and affine registration. The pre‐processed images were then segmented into gray matter, white matter, and cerebrospinal fluid components. These images were spatially normalized to a standard Montreal Neurological Institute (MNI) space using geodesic shooting registrations (Ashburner and Friston 2011). Total intracranial volume (TIV) was calculated with CAT12.

#### 2.3.7 Region of Interest (ROI) Definition

**FIGURE 1:**
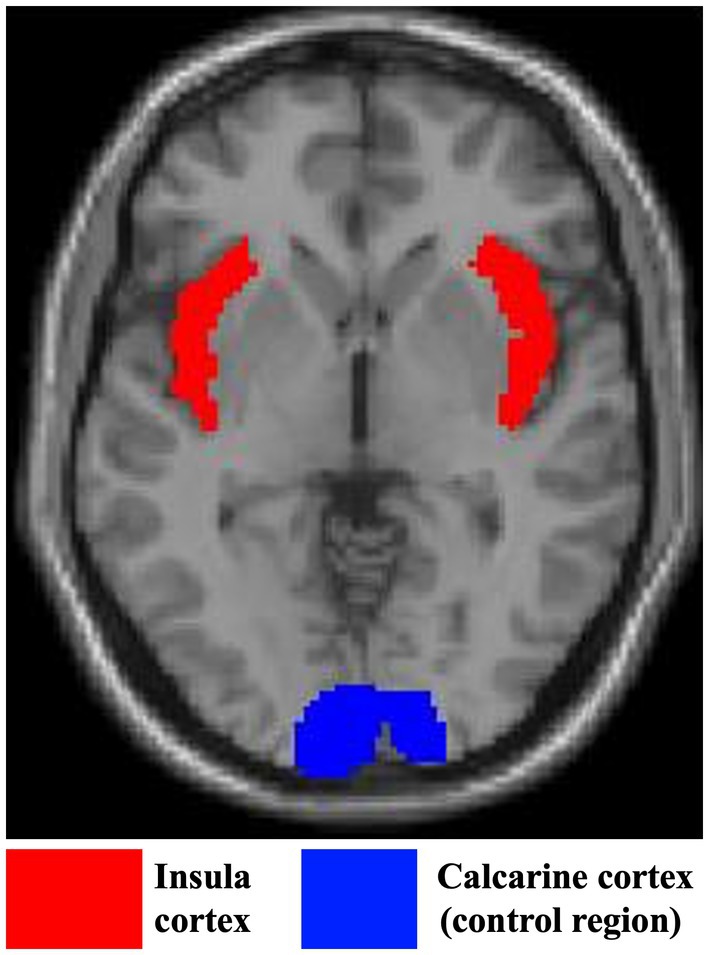
Axial view of the bilateral insula (red) and calcarine cortex (blue) regions of interest.

The total insula cortex ROI and calcarine cortex ROI were generated from the anatomical automatic labeling (AAL) system (Tzourio‐Mazoyer et al. 2002). The calcarine cortex was used as a control. It is primarily involved in processing basic visual information and is relatively preserved with aging in volume over time (Raz et al. 2010; Rehman and Al Khalili 2019). Therefore, we used the calcarine cortex to assess the specificity of insular cortex volume in relation to interoception and sleep across the adult lifespan. The definition of the ROIs is shown in Figure 1. These ROIs were then implemented in the WFU PickAtlas software toolbox (Maldjian et al. 2003). Gray matter volumes (GMV) of these ROIs were extracted using get_total.m script by Ridgway (http://www0.cs.ucl.ac.uk/staff/G.Ridgway/vbm/get_totals.m) and imported into R studio for further analyses. The ratio of the volume of each individual's ROI to their TIV was used in the analysis.

### 2.4 Data Analysis

Hierarchical multiple regression was used to test whether insula volume and age were associated with objectively measured sleep variables (sleep duration/efficiency and restlessness; see Section 2.3.3). We entered sex and calcarine gray matter volume (control region) as covariates in Block 1, followed by insula volume and age in Block 2. Sleep duration/efficiency and restlessness components were used as outcome variables in the separate models.

Hierarchical multiple regression was used to test whether insula volume and age were associated with interoceptive constructs (see Sections 2.3.1 and 2.3.2). We entered sex and calcarine gray matter volume (control region) as covariates in Block 1, followed by insula volume and age in Block 2. Interoceptive accuracy and interoceptive sensibility were used as outcome variables in the separate models.

At last, hierarchical multiple regression was used to test whether interoceptive constructs and sleep variables were related and anxiety level moderated these relationships. We entered sex as a covariate in Block 1, followed by age, interoceptive accuracy, interoceptive sensibility, and anxiety in Block 2. In Block 3, the interaction terms between interoceptive constructs and anxiety were included. Sleep duration/efficiency and restlessness component were used as outcome variables in the separate models.

Data were analyzed using R Studio. All predictors were mean‐centered prior to inclusion in the regression analyses, and no signs of multicollinearity were observed (i.e., all VIF values were below 10). We calculated 95% confidence intervals (CIs) for the unstandardized regression coefficients (*B*) in each block.

## 3 Results

### 3.1 Relationship Between the Study Variables

The results of the Pearson correlation analysis are presented in Table S1. Older age was significantly associated with smaller total insula volume (*r* = −0.61, *p* < 0.001). However, age was not related to any health variables including BMI, FMI, or HRV (all *p*s > 0.05).

### 3.2 Relationship Between Insula GMV and Sleep

The results of the hierarchical regressions investigating the relationship between total insula GMV and sleep duration/efficiency and restlessness are presented in Table 4. The findings indicated that older age predicted increased sleep duration/efficiency. Total insula GMV was not related to either sleep duration/efficiency or sleep restlessness.

**TABLE 4:** Hierarchical multiple regression with insula GMV and age predicting sleep.

	Block 1				Block 2			
	*B*	SE *B*	*β*	95% CI	*B*	SE *B*	*β*	95% CI
*Outcome variable: Sleep duration/efficiency*								
Calcarine GMV	0.019	0.086	0.027	[−0.092, 0.042]	0.115	0.091	0.161	[−0.102, 0.047]
Sex	0.224	0.167	0.161	[−0.212, 0.049]	0.191	0.169	0.138	[−0.208, 0.069]
Age					0.014	0.006	0.381*	[−0.006, 0.003]
Insula GMV					0.043	0.124	0.054	[−0.13, 0.073]
*R* ^2^	0.026				0.13			
*F* for ∆*R* ^2^	0.906				3.885*			
*Outcome variable: Sleep restlessness*								
Calcarine GMV	−0.189	0.101	−0.217	[−0.391, 0.013]	−0.157	0.112	−0.18	[−0.381, 0.066]
Sex	−0.415	0.198	−0.244*	[−0.809, 0.02]	−0.468	0.208	−0.275*	[−0.885, −0.052]
Age					0.009	0.007	0.19	[−0.006, 0.023]
Insula GMV					0.114	0.153	0.116	[−0.191, 0.419]
*R* ^2^	0.1				0.121			
*F* for ∆*R* ^2^	3.735*				0.752			

Abbreviation: GMV = gray matter volume.

* *p* < 0.05.

### 3.3 Relationship Between Insula GMV and Interoception

The results of the hierarchical regressions investigating the relationship between total insula GMV and interoceptive accuracy and sensibility are shown in Table 5. As seen in Table 5, neither total insula volume nor age predicted interoceptive accuracy. However, older age and greater total insula volume predicted greater interoceptive sensibility.

**TABLE 5:** Hierarchical multiple regression with insula GMV and age predicting interoception.

	Block 1				Block 2			
	*B*	SE *B*	*β*	95% CI	*B*	SE *B*	*β*	95% CI
*Outcome variable: Interoceptive accuracy*								
Calcarine GMV	−0.025	0.033	−0.089	[−0.391, 0.013]	−0.027	0.037	−0.098	[−0.067, 0.296]
Sex	−0.082	0.065	−0.151	[−0.809, −0.02]	−0.069	0.069	−0.127	[−0.146, 0.529]
Age					−0.001	0.002	−0.099	[0.003, 0.026]
Insula GMV					−0.029	0.051	−0.091	[−0.204, 0.29]
*R* ^2^	0.029				0.036			
*F* for ∆*R* ^2^	1.008				0.221			
*Outcome variable: Interoceptive sensibility*								
Calcarine GMV	−0.037	0.103	−0.044	[−0.243, 0.169]	0.059	0.105	0.07	[−0.151, 0.269]
Sex	−0.128	0.201	−0.078	[−0.53, 0.274]	−0.267	0.196	−0.162	[−0.659, 0.124]
Age					0.025	0.007	0.545*	[0.011, 0.038]
Insula GMV					0.291	0.144	0.304*	[0.003, 0.578]
*R* ^2^	0.008				0.174			
*F* for ∆*R* ^2^	0.255				6.565*			

Abbreviation: GMV = gray matter volume.

* *p* < 0.05.

### 3.4 The Relationship Between Interoception and Sleep, With the Moderating Effect of Anxiety

**FIGURE 2:**
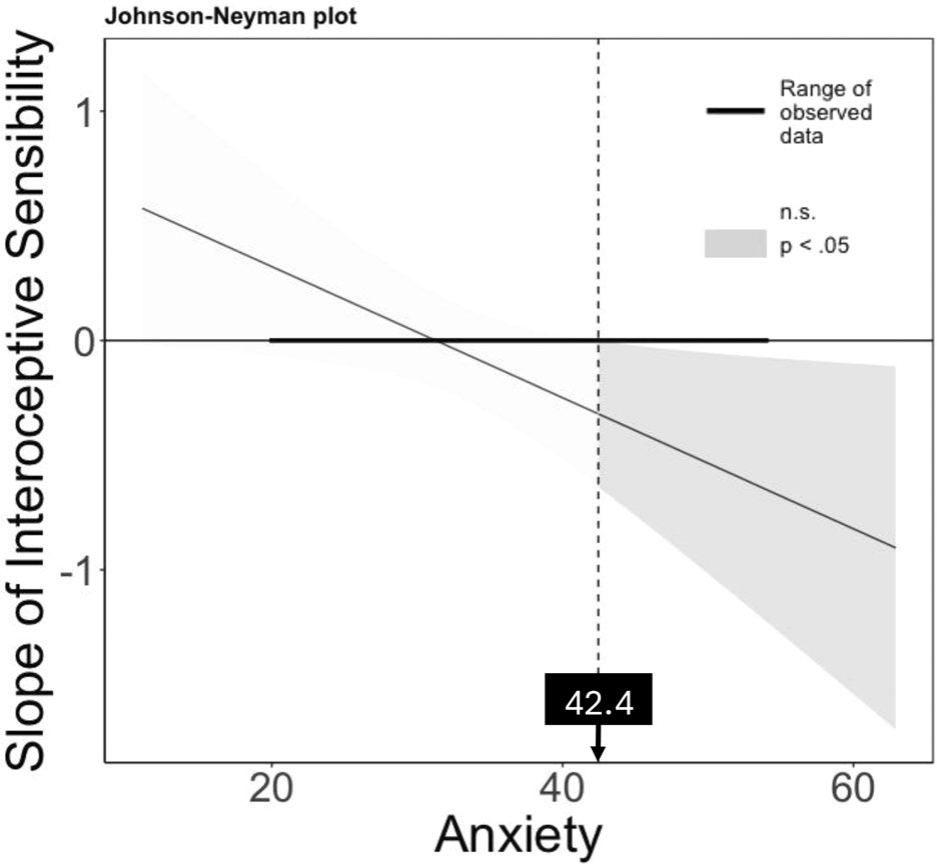
Johnson–Neyman plot of interceptive sensibility × anxiety interaction on sleep duration/efficiency.

The results of the hierarchical multiple regression investigating the relationship between interoception and sleep with moderating effects of age and anxiety are presented in Table 6. As shown in Table 6, anxiety significantly moderated the relationship between interoceptive sensibility and sleep duration/efficiency. Figure 2 illustrates the source of the interaction, showing that the negative relationship between interoceptive sensibility and sleep duration/efficiency became stronger as anxiety levels increased. Specifically, for anxiety scores above 42.46, considered a moderate‐high anxiety level (Spielberger et al. 1983; Kayikcioglu et al. 2017), greater interoceptive sensibility was associated with decreased sleep duration/efficiency. We found no significant moderating effect of age. Additionally, the results showed that higher interoceptive accuracy was linked to greater sleep restlessness, regardless of anxiety levels or age. We conducted additional hierarchical multiple regression analyses for three separate components of the sleep duration/efficiency variable to determine whether the overall effect was driven by any specific component. The interaction effects were strongest for sleep onset latency and sleep efficiency (see Table S2).

**TABLE 6:** Hierarchical multiple regression with interoception predicting sleep, with moderating effects of age and anxiety.

	Block 1				Block 2				Block 3			
	*B*	SE *B*	*β*	95% CI	*B*	SE *B*	*β*	95% CI	*B*	SE *B*	*β*	95% CI
*Outcome variable: Sleep duration/efficiency*												
Sex	0.221	0.166	0.16	[−0.109, 0.553]	0.314	0.168	0.226	[−0.022, 0.649]	0.211	0.171	0.152	[−0.131, 0.552]
Age					0.008	0.004	0.201	[−0.002, 0.017]	0.01	0.005	0.273*	[0, 0.02]
IAcc					−0.01	0.302	−0.004	[−0.613, 0.594]	−0.002	0.298	−0.001	[−0.598, 0.594]
IS					−0.045	0.107	−0.054	[−0.259, 0.169]	−0.089	0.106	−0.106	[−0.302, 0.123]
Anx					−0.023	0.01	−0.291*	[−0.043, −0.002]	−0.02	0.01	−0.261*	[−0.04, 0]
IAcc × Anx									−0.004	0.036	−0.015	[−0.054, −0.003]
IS × Anx									−0.029	0.013	−0.269*	[−0.054, −0.003]
*R* ^2^	0.026				0.17				0.235			
*F* for ∆*R* ^2^	1.785				2.779*				2.659			
*Outcome variable: Sleep restlessness*												
Sex	−0.393	0.201	−0.231	[−0.795, 0.007]	−0.414	0.203	−0.243*	[−0.819, −0.008]	−0.324	0.21	−0.191	[−0.744, 0.469]
Age					0.01	0.006	0.225	[−0.001, 0.022]	0.006	0.006	0.134	[−0.006, 0.018]
IAcc					0.882	0.365	0.281*	[0.153, 1.612]	0.966	0.366	0.307*	[0.234, 1.698]
IS					0.07	0.13	0.068	[−0.189, 0.329]	0.1	0.131	0.097	[−0.162, 0.361]
Anx					0.016	0.012	0.165	[−0.009, 0.04]	0.014	0.012	0.148	[−0.01, 0.039]
IAcc × Anx									0.069	0.044	0.195	[−0.023, 0.04]
IS × Anx									0.009	0.016	0.067	[−0.019, 0.157]
*R* ^2^	0.054				0.195				0.235			
*F* for ∆*R* ^2^	3.849				2.811*				1.629			

Abbreviations: Anx = anxiety, IAcc = interoceptive accuracy, IS = interoceptive sensibility.

* *p* < 0.05.

### 3.5 The Summary of Significant Results

**FIGURE 3:**
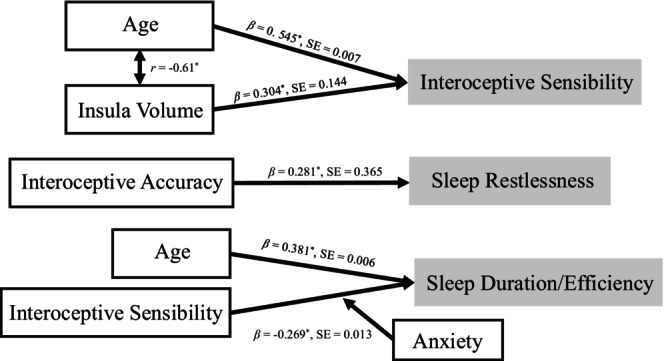
Summary of the significant results. *p < 0.05. Gray boxes indicate the outcome variables from the regression models.

The summary of significant results in the current study is shown in Figure 3. Our results indicate that older age and larger insula volume are associated with higher interoceptive sensibility. Additionally, we observed a link between greater interoceptive accuracy and increased sleep restlessness. Age also showed a relationship with sleep duration/efficiency, as older individuals tended to sleep longer and more efficiently. Interestingly, higher interoceptive sensibility was associated with decreased sleep duration/efficiency, particularly among individuals with elevated anxiety levels.

## 4 Conclusions

In this study, we examined (1) whether insula volume and age were associated with objectively measured sleep variables (sleep duration/efficiency and restlessness) and interoceptive constructs (interoceptive accuracy and sensibility), and (2) whether the relationships between interoceptive constructs and sleep variables varied as a function of anxiety levels. Greater insula volume was associated with higher interoceptive sensibility across age but was not related to sleep quality or quantity variables. Older age was related to greater interoceptive sensibility, but not to interoceptive accuracy. Interoception was related to sleep in two ways: increased interoceptive accuracy was related to greater sleep restlessness, regardless of anxiety level; and greater interoceptive sensibility was associated with decreased sleep duration/efficiency across the adult lifespan, particularly in individuals with higher anxiety. The results and their implications are discussed in the following sections.

We predicted that smaller insula volume would be related to poorer sleep, indicated by decreased sleep duration/efficiency and greater sleep restlessness. Interestingly, we did not find any significant relationship between insula volume and any of the sleep variables. These findings are inconsistent with some previous research suggesting that smaller total insula volume is linked to greater self‐reported sleep disturbances in healthy middle‐aged to older adults (Branger et al. 2016). It is possible that this discrepancy stems from the characteristics of the sample in the current study. The older adults in our sample were arguably healthier than those in the general population as age was not associated with any of the health variables including BMI, FMI, and HRV (see Table S1). Additionally, age was not associated with lower sleep quantity or quality, as reported in previous studies (as reviewed in Casagrande et al. 2022; Wai and Yu 2020). In fact, older adults in the current study (aged 60–79) had longer sleep durations (7.16 vs. 6.54 h) and higher sleep efficiency (84.72% vs. 79.94%) compared to those reported in the meta‐analysis investigating the relationship between age and actigraphy‐assessed sleep across the lifespan (Evans et al. 2021). One speculative explanation for the null findings is that the relationship between insula volume and sleep only becomes apparent in individuals with smaller insula volume/worse insula integrity or poorer sleep quality. It is also worth noting that previous studies investigating the relationship between insula integrity and sleep relied on self‐reported sleep measures (Branger et al. 2016; Li, Dong, et al. 2018; Li, Vitiello, and Gooneratne 2018). However, self‐perceptions of one's sleep are often inaccurate compared to objective measures, including actigraphy as measured here, especially in older adults (Girschik et al. 2012; Ma et al. 2021; Miner et al. 2022). Additionally, subjective sleep complaints often reflect factors in addition to sleep quality, such as somatic complaints (Schlarb et al. 2017; Zhang et al. 2012), negative affect (Chen et al. 2014; as reviewed in Fairholme and Manber 2015), and interoception, as discussed in the following section.

We hypothesized that smaller insula volume would be associated with both lower interoceptive accuracy and sensibility. Consistent with our predictions and previous research (Longarzo et al. 2021), we found that insula volume was positively related to interoceptive sensibility. The finding of a positive relationship between insula integrity and interoceptive sensibility across the adult lifespan expands on previous studies that examined this link only in young to middle‐aged adults (Longarzo et al. 2021). Interestingly, we found no significant relationship between insula volume and interoceptive accuracy, contrary to our prediction and some prior findings (Fermin et al. 2023). It might be the case that the positive relationship between insula volume and interoceptive accuracy shown in one prior study was specifically evident in middle‐aged females (i.e., Fermin et al. 2023: age range 43–62, median age 52.5). This relationship may not extend to the entire adult population or both sexes, as indicated by previous evidence of sex and age differences in interoceptive accuracy (Khalsa et al. 2008; Longarzo et al. 2021; Prentice et al. 2022; Spooner et al. 2024). Notably, we also found that older age was associated with greater interoceptive sensibility but was unrelated to interoceptive accuracy. It is possible that older individuals may believe they accurately perceive their bodily sensations, regardless of their actual ability to perceive cardiac signals under perturbation conditions. This could be due to an overconfidence tendency in responses, as shown in various cognitive domains among cognitively normal older adults (as reviewed in Spaniol and Bayen 2005). Further studies are needed to better understand how age and insula volume relate to interoceptive accuracy in a wide range of age groups and health and brain conditions.

We predicted that increased interoception would be associated with better sleep quality, based on some of the previous findings (Ewing et al. 2017; LoBrutto 2024). We also hypothesized that anxiety would moderate these relationships, such that higher levels of anxiety would weaken or even reverse the positive association. Contrary to our prediction, we found that greater interoceptive accuracy was related to increased sleep restlessness, regardless of age or anxiety levels. Greater interoceptive sensibility was associated with decreased sleep duration/efficiency, particularly at higher anxiety levels across ages. The existing literature on the connection between interoception and sleep is limited and inconsistent, with most studies focusing on younger adults or clinical populations and relying on self‐reported measures of sleep quality (Arora et al. 2021; Ewing et al. 2017; LoBrutto 2024). It is possible that the positive relationship between interoception and sleep observed in previous studies applies specifically to younger adults (Arora et al. 2021; LoBrutto 2024), especially those with subjective sleep complaints. We also found that anxiety moderates the relationship between interoception and objectively measured sleep, extending previous findings of anxiety's moderating role in the relationship between interoception and subjective sleep quality (Ewing et al. 2017). Many studies have shown a link between anxiety and interoception (as reviewed in Domschke et al. 2010; Paulus and Stein 2010; Pollatos et al. 2009; Richards et al. 2003). Individuals with anxiety tend to exhibit reduced autonomic flexibility, meaning their physiological arousal and responsiveness are less adaptive (as reviewed in Hoehn‐Saric and McLeod 2000). This often results in heightened physical arousal even at rest, which is also associated with disruptions in circadian rhythms (as reviewed in Coles et al. 2015). Previous studies have emphasized this arousal before sleep as a target for insomnia interventions (Harvey 2002), shaping one of the core principles of cognitive‐behavioral therapy for insomnia (CBT‐I; Cincotta et al. 2011; Ong et al. 2018; Sunnhed and Jansson‐Fröjmark 2015; as reviewed in Thakral et al. 2020). Given the cross‐sectional nature of this study, determining the directionality between interoception and sleep is challenging. One possibility is that heightened interoceptive accuracy and sensibility contribute to difficulties in initiating or maintaining sleep, particularly among individuals with high anxiety levels. It is also possible that autonomic dysfunction associated with chronic sleep disturbances or heightened anxiety might lead to increased interoceptive sensibility, as individuals become more sensitive to bodily sensations. Although autonomic dysfunction is typically linked to aging and poor sleep quality (as reviewed in Mather 2025; Dodds et al. 2017; Zoccoli and Amici 2020), our older participants were generally healthy and reported good sleep. It would be interesting to explore whether these relationships manifest differently in a larger and more diverse sample where older individuals may experience poorer sleep.

The current study has some limitations that should be noted. First, we only included an interoceptive accuracy measure for cardiac signals. Although this is one of the most used measures for interoceptive accuracy, interoception encompasses a wide range of bodily sensations including respiratory, nociceptive, and thermal systems (as reviewed in Khalsa et al. 2008). Therefore, future research should include multiple physiological systems when assessing interoceptive accuracy. Second, since this is a cross‐sectional study, it may not be the ideal approach for investigating changes in the interoception‐sleep link across the adult lifespan. Although we hypothesized that interoception influences sleep quality, it is possible that this relationship is bidirectional. Thus, future studies should employ a longitudinal design to better examine the directionality of the relationship between interoception and sleep. At last, our study used the VBM approach, which focuses on structural differences in the brain but does not assess functional activity.

Collectively, our findings indicate a close relationship between heightened interoception and poorer sleep quality across the adult lifespan, particularly among individuals with high anxiety levels. We also found that greater insula volume was linked to a stronger subjective belief in one's interoceptive abilities rather than to actual accuracy in detecting internal signals. Although there was no direct link between insula volume and sleep quality, it is possible that the integrity of the insula may indirectly relate to sleep quality through its association with interoceptive sensibility. These findings may have implications for sleep interventions focused on mindfulness including those that enhance interoception (as reviewed in Chan et al. 2022; also see Gibson 2019). This is particularly important for individuals with anxiety and older adults, who are more likely to experience heightened interoceptive sensibility. Tailored interventions should take into account the unique characteristics of interoceptive constructs and individual anxiety levels.

## Author Contributions

**Ahhyun Seo:** conceptualization, formal analysis, investigation, methodology, visualization, writing – original draft, writing – review and editing. **Risako Nishiyama:** formal analysis, investigation, writing – original draft, writing – review and editing. **Kyoungeun Lee:** conceptualization, data curation, project administration, resources. **Audrey Duarte:** conceptualization, data curation, project administration, supervision, validation, writing – review and editing.

## Conflicts of Interest

The authors declare no conflicts of interest.

## Supporting information


**Data S1:** psyp70120‐sup‐0001‐supinfo.docx.

## Data Availability

Data available on request due to privacy/ethical restrictions.
