# Supplementary material for: Factors Linking Interoception and Sleep Across the Adult Lifespan
Source: Psychophysiology. 2025 Aug 6;62(8):e70120. doi: 10.1111/psyp.70120 (PMC12326225; doi:10.1111/psyp.70120)
Supplement: Supplementary file 1 — Data S1: psyp70120‐sup‐0001‐supinfo.docx. [file PSYP-62-e70120-s001.docx]

**Supplementary Table 1.** Correlations among study variables.

| Variable | 1 | 2 | 3 | 4 | 5 | 6 | 7 | 8 | 10 | 11 | 12 | 13 | 14 |
| --- | --- | --- | --- | --- | --- | --- | --- | --- | --- | --- | --- | --- | --- |
| 1. Age | - |  |  |  |  |  |  |  |  |  |  |  |  |
| 2. Sex | 0.05 | - |  |  |  |  |  |  |  |  |  |  |  |
| 3. IAcc | -0.01 | -0.15 | - |  |  |  |  |  |  |  |  |  |  |
| 4. IS | 0.32^*^ | -0.08 | 0.21 | - |  |  |  |  |  |  |  |  |  |
| 5. Not-Worrying | 0.27 | -0.16 | 0.05 | 0.26^*^ | - |  |  |  |  |  |  |  |  |
| 6. Not-Distracting | -0.05 | -0.04 | -0.01 | -0.15 | -0.08 | - |  |  |  |  |  |  |  |
| 7. Sleep Duration | 0.29^*^ | 0.16 | -0.04 | 0.09 | 0.02 | 0.16 | - |  |  |  |  |  |  |
| 8. Sleep Restlessness | 0.18 | -0.23 | 0.32^*^ | 0.16 | -0.02 | -0.13 | -0.28^*^ | - |  |  |  |  |  |
| 10. Insula GMV | -0.61^*^ | 0.20 | -0.09 | -0.04 | -0.08 | -0.01 | -0.10 | -0.12 | - |  |  |  |  |
| 11. Anxiety | -0.32^*^ | 0.28^*^ | -0.05 | -0.34^*^ | -0.43^*^ | -0.23 | -0.27^*^ | -0.01 | 0.27^*^ | - |  |  |  |
| 12. BMI | 0.11 | -0.05 | -0.11 | -0.10 | 0.05 | -0.10 | 0.17 | -0.12 | -0.35^*^ | -0.12 | - |  |  |
| 13. FMI | 0.19 | 0.41^*^ | -0.14 | -0.11 | -0.03 | -0.10 | 0.23 | -0.15 | -0.30^*^ | 0.01 | 0.91^*^ | - |  |
| 14. HRV | 0.09 | -0.03 | -0.03 | 0.03 | 0.11 | 0.10 | -0.04 | 0.07 | -0.04 | -0.14 | 0.17 | 0.09 | - |

*Note.* ^*^ *p* < 0.05. Sex (0 = male, 1 = female). IAcc = Interoceptive Accuracy, IS = Interoceptive Sensitivity (The general factor of MAIA). GMV = Gray Matter Volume.

*Note.* The ratio of the gray matter volume of each individaul’s insula cortex to their TIV used as the index of insula GMV.

**Supplementary Table 2.** Hierarchical multiple regression with interoception predicting each component of the sleep duration/efficiency variable, with moderating effects of age and anxiety.

|  | Block 1 | | | | Block 2 | | | | | | Block 3 | | | |
| --- | --- | --- | --- | --- | --- | --- | --- | --- | --- | --- | --- | --- | --- | --- |
|  | *B* | *SE B* | *β* | 95% CI | *B* | *SE B* | | | *β* | 95% CI | *B* | *SE B* | *β* | 95% CI |
| Outcome variable: Sleep Efficiency | | | | | | | | | | | | | | |
| Sex | -0.235 | 0.212 | -0.133 | [-0.657, 0.188] | -0.322 | 0.217 | | | -0.183 | [-0.756, 0.111] | -0.151 | 0.217 | -0.086 | [-0.584, 0.283] |
| Age |  |  |  |  | 0.005 | 0.006 | | | 0.115 | [-0.007, 0.018] | 0.012 | 0.006 | 0.242 | [-0.001,  0.024] |
| IAcc |  |  |  |  | -0.42 | 0.39 | | | -0.129 | [-1.199, 0.359] | -0.486 | 0.378 | -0.149 | [-1.242, 0.271] |
| IS |  |  |  |  | -0.144 | 0.139 | | | -0.135 | [-0.421, 0.133] | -0.21 | 0.135 | -0.196 | [-0.48, 0.06] |
| Anx |  |  |  |  | -0.03 | 0.013 | | | -0.303^*^ | [-0.056, -0.004] | -0.026 | 0.013 | -0.267^*^ | [-0.052,  -0.001] |
| IAcc x Anx |  |  |  |  |  |  | | |  |  | -0.064 | 0.045 | -0.175 | [-0.155,  0.027] |
| IS x Anx |  |  |  |  |  |  | | |  |  | -0.034 | 0.016 | -0.249^*^ | [-0.066, -0.001] |
| R^2^ | 0.018 | | | | 0.143 | | | | | | 0.238 | | | |
| F for ∆R^2^ | 1.226 | | | | 2.347 | | | | | | 3.863^*^ | | | |
| Outcome variable: Onset Latency | | | | | | | | | | | | | | |
| Sex | 0.104 | 0.184 | 0.068 | [-0.263, 0.471] | 0.115 | 0.196 | | | 0.076 | [-0.276, 0.507] | 0.048 | 0.2 | 0.032 | [-0.351, 0.447] |
| Age |  |  |  |  | -0.008 | 0.006 | | | -0.201 | [-0.019, 0.03] | -0.009 | 0.006 | -0.207 | [-0.02,  0.003] |
| IAcc |  |  |  |  | -0.433 | 0.352 | | | -0.154 | [-1.136, 0.27] | -0.51 | 0.348 | -0.182 | [-1.21, 0.186] |
| IS |  |  |  |  | 0.059 | 0.125 | | | 0.064 | [-0.191, 0.309] | 0.095 | 0.124 | 0.103 | [-0.154, 0.343] |
| Anx |  |  |  |  | 0.0001 | 0.012 | | | 0.001 | [-0.024, 0.024] | -0.002 | 0.012 | -0.022 | [-0.025,  0.021] |
| IAcc x Anx |  |  |  |  |  |  | | |  |  | -0.05 | 0.042 | -0.157 | [-0.133,  0.034] |
| IS x Anx |  |  |  |  |  |  | | |  |  | 0.032 | 0.015 | 0.272^*^ | [0.002, 0.061] |
| R^2^ | 0.005 | | | | 0.06 | | | | | | 0.131 | | | |
| F for ∆R^2^ | 0.32 | | | | 2.181 | | | | | | 2.547 | | | |
| Outcome variable: TST | | | | | | | | | | | | | | |
| Sex | -0.358 | 0.24 | -0.178 | [-0.838, 0.121] | -0.523 | | 0.236 | -0.26^*^ | | [-0.993, -0.052] | -0.447 | 0.247 | -0.222 | [-0.94, 0.046] |
| Age |  |  |  |  | 0.01 | | 0.007 | 0.174 | | [-0.004, 0.023] | 0.011 | 0.007 | 0.207 | [-0.003,  0.026] |
| IAcc |  |  |  |  | -0.122 | | 0.423 | -0.033 | | [-0.968, 0.723] | -0.109 | 0.430 | -0.029 | [-0.969, 0.751] |
| IS |  |  |  |  | 0.104 | | 0.15 | 0.085 | | [-0.196, 0.405] | 0.1071 | 0.154 | 0.058 | [-0.236, 0.378] |
| Anx |  |  |  |  | -0.036 | | 0.014 | -0.319^*^ | | [-0.064, -0.008] | -0.034 | 0.014 | -0.303^*^ | [-0.063,  -0.005] |
| IAcc x Anx |  |  |  |  |  | |  |  | |  | 0.002 | 0.052 | 0.006 | [-0.101,  0106] |
| IS x Anx |  |  |  |  |  | |  |  | |  | -0.022 | 0.018 | -0.145 | [-0.059, 0.145] |
| R^2^ | 0.032 | | | | 0.227 | | | | | | 0.246 | | | |
| F for ∆R^2^ | 2.225 | | | | 4.047^*^ | | | | | | 1.276 | | | |

Note. ^*^*p* < 0.05. IAcc = Interoceptive Accuracy. IS = Interoceptive Sensibility. Anx = Anxiety.
